# Involvement of oxidative stress and mitochondrial mechanisms in air pollution-related neurobiological impairments

**DOI:** 10.1016/j.ynstr.2019.100205

**Published:** 2019-12-19

**Authors:** Ankita Salvi, Hesong Liu, Samina Salim

**Affiliations:** Department of Pharmacological and Pharmaceutical Sciences, College of Pharmacy, University of Houston, Houston, TX, 77204, USA

**Keywords:** Traffic pollution, Toxicological stress, Behavioral health, Oxidative stress, Mitochondria

## Abstract

**Background:**

Vehicle exhaust emissions are known to be significant contributors to physical and psychological stress. Vehicle exhaust-induced stress and associated respiratory and cardiovascular complications are well-known, but the impact of this stress on the brain is unclear. Simulated vehicle exhaust exposure (SVEE) in rats causes behavioral and cognitive deficits. In the present study, the underlying mechanisms were examined. Our postulation is that SVEE, a simulation of physiologically relevant concentrations of pro-oxidants (0.04% carbon dioxide, 0.9 ppm nitrogen dioxide, 3 ppm carbon monoxide) creates a toxic stress environment in the brain that results in an imbalance between production of reactive oxygen species and the counteracting antioxidant mechanisms. This impairs mitochondrial function in the high bioenergetic demand areas of the brain including the hippocampus (HIP), amygdala (AMY) and the prefrontal cortex (PFC), disrupting neuronal network, and causing behavioral deficits. Mitochondria-targeted antioxidant Mito-Q protects against these impairments.

**Methods:**

Sprague Dawley rats were provided with Mito-Q (250 μM) in drinking water for 4 weeks followed by SVEE 5 h/day for 2 weeks, followed by behavioral and biochemical assessments.

**Results:**

SVEE resulted in anxiety- and depression-like behavior, accompanied with increased oxidative stress, diminished antioxidant response and mitochondrial impairment reflected from electron transport chain (ETC) disruption, reduced oxygen consumption, low adenosine tri-phosphate (ATP) synthesis and an alteration in the mitochondrial biochemical dynamics assessed via protein expression profiles of mitochondrial fission marker, dynamin-related protein-1 and fusion markers, mitofusin-1/2 in the HIP, AMY and the PFC. Mito-Q treatment prevented SVEE-induced behavioral deficits, attenuated rise in oxidative stress and also prevented SVEE-induced mitochondrial impairment.

**Conclusion:**

This study demonstrates a causal mechanism mediating SVEE-induced behavioral deficits in rats. We further established that SVEE is a toxicological stressor that induces oxidative stress and results in mitochondrial impairment, which by disrupting neural circuitry impairs cognitive and behavioral functions.

## Highlights

• Simulated vehicle exhaust exposure is a source of toxicological stress.
• Prolonged exposure leads to behavioral deficits and elevated oxidative stress.
• Oxidative stress elevation triggers mitochondrial impairment in the brain.
• Mito-Q prevents exhaust-associated behavioral and biochemical alterations.

## Graphical abstract

**Figure:**
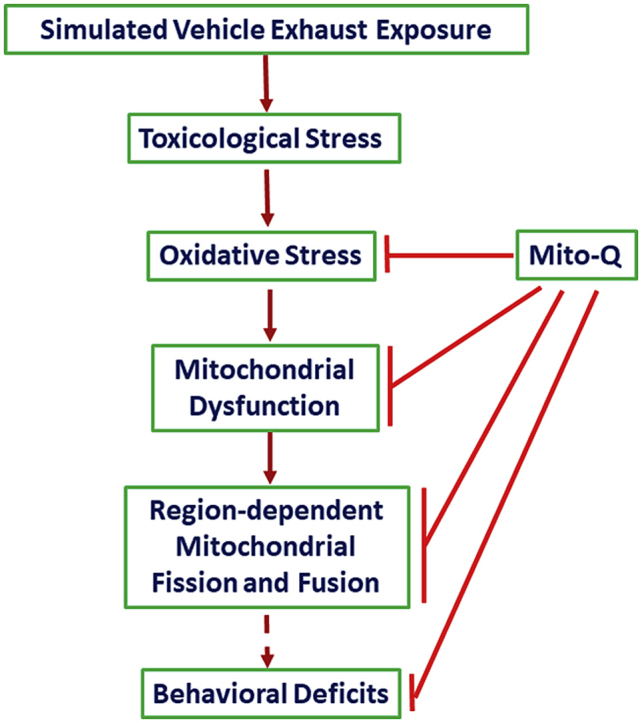

## Abbreviations

SVEE: simulated vehicle exhaust exposure
CO_2_: carbon dioxide
NO_2_: nitrogen dioxide
CO: carbon monoxide
HIP: hippocampus
AMY: amygdala
PFC: prefrontal cortex
ETC: electron transport chain
ATP: adenosine triphosphate
DE: diesel exhaust
CON + VEH: control + vehicle
CON + MITO-Q: control + Mito-Q
EXP + VEH: exposure + vehicle
EXP + MITO-Q: exposure + Mito-Q
OFT: open field test
EPM: elevated plus maze
LD: light-dark
MB: marble burying
FST: forced swim test
MFN-1/2: mitofusin-1/2
DRP-1: dynamin-related protein-1
SOD: superoxide dismutase
ELISA: enzyme-linked immunosorbent assay
JC-1: tetraethylbenzimidazolylcarbocyanine iodide

## 1 Introduction

Air pollution, a result of increased urbanization, is a serious environmental hazard and a major health risk factor. In fact, the association between air pollution and morbidity and mortality caused by respiratory and cardiovascular diseases is well established (Gill et al., 2011; Brook and Rajagopalan, 2007). Emerging evidence suggests that air pollution contributes to physical and psychological stress (Sass et al., 2017; Thomson, 2019). Stress is an inclusive term used for events that disrupt homeostasis in the body; and could be physical (trauma, environmental pollutants, illness), psychological (emotions, fear) or a combination of both (Selye, 1956; Brook and Rajagopalan, 2007; Bremner, 2006). Toxic constituents of vehicle exhaust such as carbon monoxide, particulate matter, organic hydrocarbons act as physical stressors, altering molecular pathways and resulting in deleterious health effects (Frank and Tankersley, 2002; Brunekreef and Holgate, 2002). Furthermore, continuous exposure to vehicle exhaust emissions is known to act as an emotional stressor and may negatively affect the central nervous system (CNS), contributing to psychiatric ailments (Calderon-Garciduenas et al., 2002; Genc et al., 2012; Costa et al. 2014, 2017; Sass et al., 2017). In this context, human epidemiological studies have shown that air pollution and vehicle exhaust are associated with decreased cognitive functions, and depressive symptoms, as well as neurodegenerative and neurodevelopmental pathologies (Calderon-Garciduenas et al. 2004, 2010, 2011, 2012; Ranft et al., 2009; Fonken et al., 2011; Power et al., 2011; Weuve et al., 2012; Guxens and Sunyer, 2012; Levesque et al., 2011). It is believed that individuals living and working in areas of heavy vehicle traffic have high susceptibility to develop anxiety, depression and poor cognition (van Donkelaar et al., 2015). Animal studies have provided further insights into this area. For instance, diesel exhaust (DE) exposure in mice was reported to alter locomotor activity and impair spatial learning and memory (Yokota et al., 2009; Hougaard et al., 2009; Suzuki et al., 2010; Win-Shwe et al., 2008). Exposure to vehicle exhaust emissions is known to trigger stress response in the body that activates the hypothalamic-pituitary-adrenal (HPA) axis (Thomson, 2019). Activation of HPA axis initiates downstream oxidative stress and neuroinflammatory pathways, and have been considered to be one of the potential links between traffic-related air pollution and adverse CNS effects (Brook et al., 2010; Lodovici and Bigagli, 2011; Anderson et al., 2012; Calderon-Garciduenas et al., 2008; Costa et al., 2004; MohanKumar et al., 2008; Kraft and Harry, 2011; Win-Shwe and Fujimaki, 2011; Block et al., 2004; Roque et al., 2016; Thomson, 2019). In spite of growing information on mechanisms underlying vehicle exhaust associated detrimental health effects, the exact mechanism mediating air pollution-associated CNS effects is unclear.

Several important points emerge from the evidence cited above, *first*, traffic-related air pollution generates neurotoxicity within the CNS. *Second*, traffic-related air pollution potentially alters neurobehavioral function. In our recent publication (Salvi et al., 2017), we directly examined the relationship between oxidative stress and behavioral functions in rats using a simulated vehicle exhaust exposure (SVEE) model. Sprague Dawley (SD) rats were exposed to a mixture comprising of pro-oxidant constituents of vehicle exhaust such as carbon dioxide (CO_2_), carbon monoxide (CO) and nitrogen dioxide (NO_2_) (Westerholm and Egeback, 1994). SVEE exposed rats developed significant behavioral and cognitive deficits (Salvi et al., 2017).This intriguing report has prompted us to ask several questions; why SVEE causes behavioral and cognitive impairments in rats? What are the underlying neurobiological mechanisms that cause these deficits? Are SVEE-induced impairments preventable? In this article, we investigate the link between SVEE-induced behavioral impairments and oxidative stress-associated mechanisms and explore potential interventions that could prevent these SVEE-associated adverse effects.

## 2 Theory

Our postulation is that SVEE constituents, by acting as a toxicological stressors, elevate oxidative stress levels in the brain. And, oxidative stress by engaging mitochondrial mechanisms possibly alter neuronal circuitry, eventually resulting in behavioral impairments. Also, interventions that limit oxidative stress, such as mitochondria-targeted antioxidant, Mito-Q prevent occurrence of SVEE-induced impairments.

## 3 Materials and methods

Following 1 week of acclimatization period, rats were administered water/Mito-Q dissolved in drinking water for 4 weeks. This was followed by a physiologically relevant dose of SVEE (0.04% CO_2_, 0.9 ppm NO_2_ and 3 ppm CO) 5 h/day for 2 continuous weeks. Next, a comprehensive behavioral analysis was performed to assess anxiety-like and depression-like behavior. Rats were then sacrificed, and an extensive biochemical analysis was performed on three brain regions namely, pre-frontal cortex (PFC), hippocampus (HIP) and amygdala (AMY) to determine level of SVEE-induced oxidative stress and mitochondrial impairment. The experimental design is depicted in Supplementary fig. 1.

### 3.1 Experimental design

Adult male SD rats (225–250 g, 10–12 weeks old) were divided into 4 groups: *Control *+*Vehicle (CON + VEH):* vehicle (water) pre-treated rats exposed to normal air; *Control + Mito-Q (CON + MITO-Q):* Mito-Q pre-treated rats exposed to normal air; *Exposure + Vehicle (EXP + VEH):* vehicle pre-treated rats exposed to simulated vehicle exhaust; *Exposure + Mito-Q (EXP + MITO-Q):* Mito-Q pre-treated rats exposed to simulated vehicle exhaust.

### 3.2 Simulated vehicle exhaust exposure model

Adult male SD rats (225–250 g) were housed in standard cages in the climate-controlled rodent housing facility on a 12-h light/dark cycle with *ad libitum* food and water. Experiments were conducted in accordance with the NIH guidelines using protocols approved by the University of Houston Animal Care and Use Committee. The Simulated Vehicle Exhaust Exposure (SVEE) apparatus consisted of two cages capable of housing 3–5 rats per cage (CH Technologies, NJ, USA). Number of rats housed per cage in the rodent housing facility was equal to number of rats housed per chamber of the apparatus during exposure. A simulated mixture of pro-oxidant gases was obtained from Scott Specialty Gases (SCOTTTM). The composition of mixture (0.04% CO_2_, 0.9 ppm NO_2_ and 3 ppm CO in air) was based upon level of pro-oxidants present in areas of heavy vehicular traffic (Win-Shwe et al., 2012). This mixture was devoid of any particulate matter or hydrocarbons. Each cage was fitted with a tubing system to allow inflow of the simulated mixture into the chamber and its outflow out of the chamber in order to maintain a continuous circulation. A flow regulator displayed the flow rate of the gaseous mixture entering the chambers (Supplementary fig. 2). The (EXP + VEH) and (EXP + MITO-Q) groups of rats were exposed to simulated vehicle exhaust in this apparatus 5 h daily for 2 weeks in which (CON + VEH) and (CON + MITO-Q) groups were exposed to normal air (used as base for the exhaust mixture) for the same duration.

### 3.3 Mito-Q treatment

*Mito-Q* or mitoquinone mesylate was obtained as a gift from Dr. Michael Murphy, Mitochondria Research Center-Dunn Human Nutrition Unit, Antipodean Pharmaceuticals, Cambridge, UK. The drug was provided as a red powder and was stored at room temperature, protected from light upon its receipt. A 250 μM solution of Mito-Q in tap water was prepared and fed orally *ad libitum* to the rats during the course of the treatment (4 weeks). The solution was prepared fresh daily and supplied to rats in light-protected amber bottles. The dose was chosen based on the optimum and well-tolerated dose of Mito-Q as reported in previously published studies (Rodriguez-Cuenca et al., 2010). Vehicle rats received normal tap water provided *ad libitum* during the course of Mito-Q treatment.

### 3.4 Quantification of Mito-Q in the brain

In order to confirm the amount of Mito-Q that reached the brain following oral administration, liquid chromatography-mass spectrometry (LC-MS) experiments were performed on whole brain samples as published (Rodriguez-Cuenca et al., 2010). Following 4 weeks of Mito-Q/vehicle treatment, few rats per group were sacrificed for LC-MS quantification. The sacrifice was performed on *day 35* as indicated in Supplementary fig. 1. The rats sacrificed belonged to either of the 2 groups: vehicle (VEH): rats provided with water for 4 weeks, treated group (MitoQ): rats provided with Mito-Q for 4 weeks. Following sacrifice, brain tissues were isolated and samples (∼100 mg wet weight) were homogenized and spiked with 20 pmol of an internal standard (IS), d_15_-Mito-Q (obtained as a gift from Dr. Michael Murphy, Antipodean Pharmaceuticals, Cambridge, UK) to prepare standard and sample solutions as previously published (Vollert et al., 2011). An LC-MS method was developed and validated in compliance with US Food and Drug Administration Guidance for Industry: Bioanalytical Method Validation. The linearity, sensitivity and selectivity of the method was confirmed by plotting a standard curve followed by quantification of Mito-Q in the VEH and MITO-Q brain samples. All LC-MS experiments were performed by Dr. Weiqun Wang, PPS Dept. College of Pharmacy, University of Houston. The experiments were performed at 40 °C using a Luna 5μ Phenyl–Hexyl column (2 × 50 mm, 5 μM) from Phenomenex, Torrance, CA. The mobile phase consisted of 0.1% formic acid in water (buffer A) and 0.1% formic acid in acetonitrile (buffer B) delivered as a linear gradient as follows: 0–2 min, 5% B; 2–5 min, 5–95% B; 5–10 min, 95% B; 10–11 min, 95–5% B; 11–18 min, 5% B. The flow rate was 250 μl/min. An inline divert valve was used to divert the eluent away from the mass spectrometer from 0 to 3 min and 12–18 min of the acquisition. Electrospray ionization was performed in positive ion mode. MitoQ (m/z = 583.3) was analyzed by multiple reaction monitoring (MRM) m/z 583.3 > 441.3.

### 3.5 Behavioral analysis

All behavioral tests were conducted in the order as indicated in Supplementary fig. 1. The experimenter was blinded to treatment.

#### 3.5.1 Anxiety-like behavior tests

The anxiety-like behavior tests comprised of open-field test which was followed by elevated-plus maze, light dark and marble burying tests as previously published by us (Patki et al., 2015; Vollert et al., 2011; Solanki et al., 2015; Salim et al., 2011; Allam et al., 2013).

##### 3.5.1.1 Open field test (OFT)

The OFT apparatus consisted of an open arena (17.5″ × 17.5″) surrounded by transparent plexiglass walls. Each rat was placed in the arena for 15 min and allowed to move freely in the arena. Time spent in the center of the arena was recorded using infrared sensors of the apparatus. Less time spent in the center of the arena of the OFT apparatus was considered an indication of anxiety-like behavior (Patki et al., 2013).

##### 3.5.1.2 Elevated plus maze (EPM) test

The apparatus consisted of two open and two closed arms (10 cm × 50 cm) that intersected to create a plus shape at an elevation of about 60 cm from the groundEach rat was placed at the intersection of the open and closed arms of the maze and allowed to explore the maze for 5 min. Reduced time spent by a rat in the open arm of the EPM and less number of crossings from the closed arm into the open arm is an indication of anxiety-like behavior (Solanki et al., 2015).

##### 3.5.1.3 Light-dark (LD) test

The LD test apparatus involved a box that consisted of two compartments: a lit compartment (27 × 27 × 27 cm) and a dark compartment (27 × 18 × 27 cm) separated by a single partition with an opening (7 × 7 cm) to facilitate inter-compartment movement. Each rat was placed in the lit compartment and was given 5 min to explore both compartments. Less time spent in the lit compartment and less number of crossings to the lit compartment suggests increase in anxiety-like behavior (Solanki et al., 2015).

##### 3.5.1.4 Marble burying (MB) test

In this test, each rat was placed in a cage with bedding for 10 min. The rat was allowed to explore the cage and acclimatized for 10 min. After 10 min, the rat was taken out of the cage and opaque glass marbles were evenly spaced on the bedding of the cage. The rat was then placed in the marble containing cage and allowed to explore the cage for another 30 min. Marbles are perceived as foreign elements by the rat. Highly anxious rats have a tendency to engage in a digging behavior when exposed to stressful situations such as presence of foreign elements in a familiar environment. More marbles buried by rats, higher the anxiety-like behavior (Deacon, 2006).

#### 3.5.2 Depression-like behavior test

Forced swim test (FST) was used to assess depression-like behavior in rats. This test comprised of a tank (24 cm in diameter and 30 cm high) filled with water (25 °C). Each rat was dropped in the tank and their mobility (immobility versus active behavior) was recorded for 5 min (no pre-test phase) Immobile time spent by the rat in the water filled cylindrical tank of FST is an indication of high depression-like behavior (Solanki et al., 2015; Salvi et al., 2017).

### 3.6 Biochemical analysis

#### 3.6.1 Brain tissue homogenization and protein estimation

Pre-frontal cortex (PFC), hippocampus and amygdala were dissected out (Paxinos and Watson, 1998), and the tissues were homogenized as published (Salim and Dessauer, 2004). Protein concentration of the lysates was estimated using microBCA assay kit from Pierce (Pierce, Rockford, IL).

#### 3.6.2 Oxidative stress parameters

##### 3.6.2.1 8-Isoprostane levels

The level of 8-isoprostane was measured using an ELISA kit (cat # 516351, Cayman Chemical, MI). Oxidative stress and subsequent increase in free radicals leads to oxidation of tissue phospholipids, generating isoprostanes, a marker of oxidative stress (Valko et al., 2007).

##### 3.6.2.2 Total antioxidant capacity (TAC)

Total antioxidant capacity was measured using TAC Assay kit (cat# MAK187, Sigma-Aldrich, MO) per manufacturer's instructions. This kit estimates the capacity of the total antioxidants in the sample to convert Cu^2+^ to its reduced form, Cu^+^ which chelates with a colorimetric probe, giving a broad absorbance peak at ∼570 nm.

##### 3.6.2.3 Protein expression of antioxidant enzyme, superoxide dismutase (SOD)

Protein expression of manganese superoxide dismutase (Mn SOD) was determined with western blotting as previously published by us (Solanki et al., 2015). Following overnight incubation with primary antibody (dilution-1:1000, cat# 06–984, Millipore, CA) at 4 °C, immunoreactive bands were detected by goat anti-rabbit horseradish peroxidase-conjugated secondary antibody (1:2000, cat# 7074S, Cell Signaling Technology, Danvers, MA). Chemiluminescence was detected by Alpha Ease FC 4.0 (Alpha Innotech Corp., San Leandro, CA) and densitometrically quantified using Fluorochem FC8800 software. The protein was normalized to β-actin loading control (sc-47778, 1:1000, Santa Cruz Biotechnology, Santa Cruz, CA.

##### 3.6.2.4 SOD activity assay

SOD Enzyme Activity was measured using an assay kit (cat# 706002 Cayman, MI) as per manufacturer's instructions. The kit uses tetrazolium salt for detection of superoxide radicals generated by xanthine oxidase and hypoxanthine. The salt converts into a dye in the presence of superoxides giving absorbance at 440–460 nm.

#### 3.6.3 Mitochondrial parameters

Mitochondrial parameters such as mitochondrial oxygen consumption, ATP synthesis, mitochondrial membrane potential and expression of fission fusion markers were assessed in mitochondrial isolates of the PFC, hippocampus and amygdala. Mitochondria were isolated from brain homogenates using mitochondria isolation kit (cat# ab110168, Abcam, MA).

##### 3.6.3.1 Mitochondrial oxygen consumption

Mitochondrial oxygen consumption was measured by high resolution respirometry using Oroboros Oxygraph series D and Datlab software (Oroboros Instruments, Innbruck, Austria). 1 μg/ml of mitochondria isolates were added to 2 ml-chambers containing respiration media (0.5 mM egtazic acid, 3 mM magnesium chloride, 60 mM lactobionic acid, 20 mM taurine, 10 mM monopotassium phosphate, 20 mM hydroxyethyl piperazineethanesulfonic acid (HEPES), 110 mM D-sucrose and 1 g/l bovine serum albumin and respiration was measured at 37 °C using routine mitochondrial respiration, corrected for residual oxygen consumption. Datlab software was used to quantitate oxygen consumption (Santidrian et al., 2013).

##### 3.6.3.2 ATP determination

ATP synthesis was measured using an ATP Determination Kit (cat# A22066, Molecular probes, OR), which uses a recombinant firefly luciferase enzyme and its substrate D-luciferin to produce light in the presence of ATP, which is measured at an emission wavelength of 560 nm.

##### 3.6.3.3 Mitochondrial membrane potential

Mitochondrial membrane potential was measured using a kit-based, assay (cat # 600880, Cayman, MI). The assay utilizes a JC-1 dye which forms J-aggregates (Ex/Em = 560/590 nm) or J-monomers (Ex/Em = 485/535 nm) after entering the mitochondria. The ratio of J-aggregate to monomer intensity indicates the mitochondrial health and depolarization of mitochondrial membrane potential. Lower the ratio, lower the membrane potential.

##### 3.6.3.4 Mitochondrial fission and fusion

Protein expression of mitochondrial fission marker, dynamin-related protein-1 (DRP-1); and fusion markers, mitofusin-1/2 (Mfn-1/2) was determined using Western blot analysis as described before (Solanki et al., 2015). Antibody dilutions: DRP-1 (ab156951, 1:1000), Mfn-1 (ab57602, 1:500) and Mfn-2 (ab56889, 1:500) were purchased from Abcam (Cambridge, MA). Anti-Secondary antibody (1:2000, Cell Signaling Technology, Danvers, MA) was used. β-actin was used as a loading control (sc-47778, 1:1000, Santa Cruz Biotechnology, Santa Cruz, CA).

### 3.7 Statistical analysis

All values were expressed as mean ± SEM. Significance was determined by 1-way ANOVA with subsequent Tukey's posthoc test (GraphPad Software, Inc. San Diego, CA). A value of p < 0.05 was considered significant.

## 4 Results

### 4.1 Quantification of Mito-Q in the brain using LC-MS

**Fig. 1:**
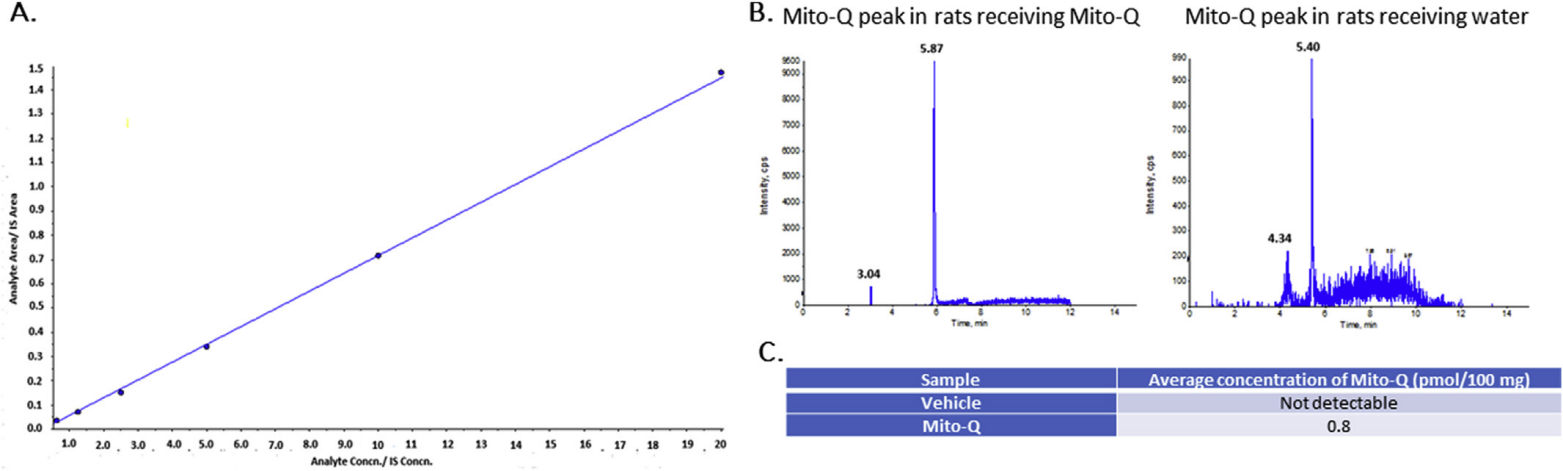
**LC-MS estimation of Mito-Q in rat brain.** (A) A linear curve with an equation of y = 0.0733x – 0.0161, r^2^ = 0.9993 was plotted with x-axis representing ratio of analyte (Mito-Q) concentration to IS (d_15_-Miito-Q) concentration and y-axis representing ratio of analyte (Mito-Q) area to IS (d_15_-Miito-Q) area. (B) Retention time of d_15_-Miito-Q (first peak) and Mito-Q (second peak) have been listed along the peaks. (C) Tabular representation of average Mito-Q concentration. n = 5 rats/group.

LC-MS experiments indicated that Mito-Q was present in picomolar concentrations in the rats that received oral Mito-Q treatment. Rats that received normal drinking water did not show detectable Mito-Q levels. Fig. 1C enlists the quantified levels of Mito-Q in VEH and Mito-Q rats. The LC-MS method was found to be linear, sensitive and reproducible. Standard curve obtained, and chromatograms of Mito-Q and d_15_-Mito-Q obtained following LC-MS are depicted in Fig. 1.

### 4.2 Behavioral analysis

#### 4.2.1 Anxiety-like behavior

**Fig. 2:**
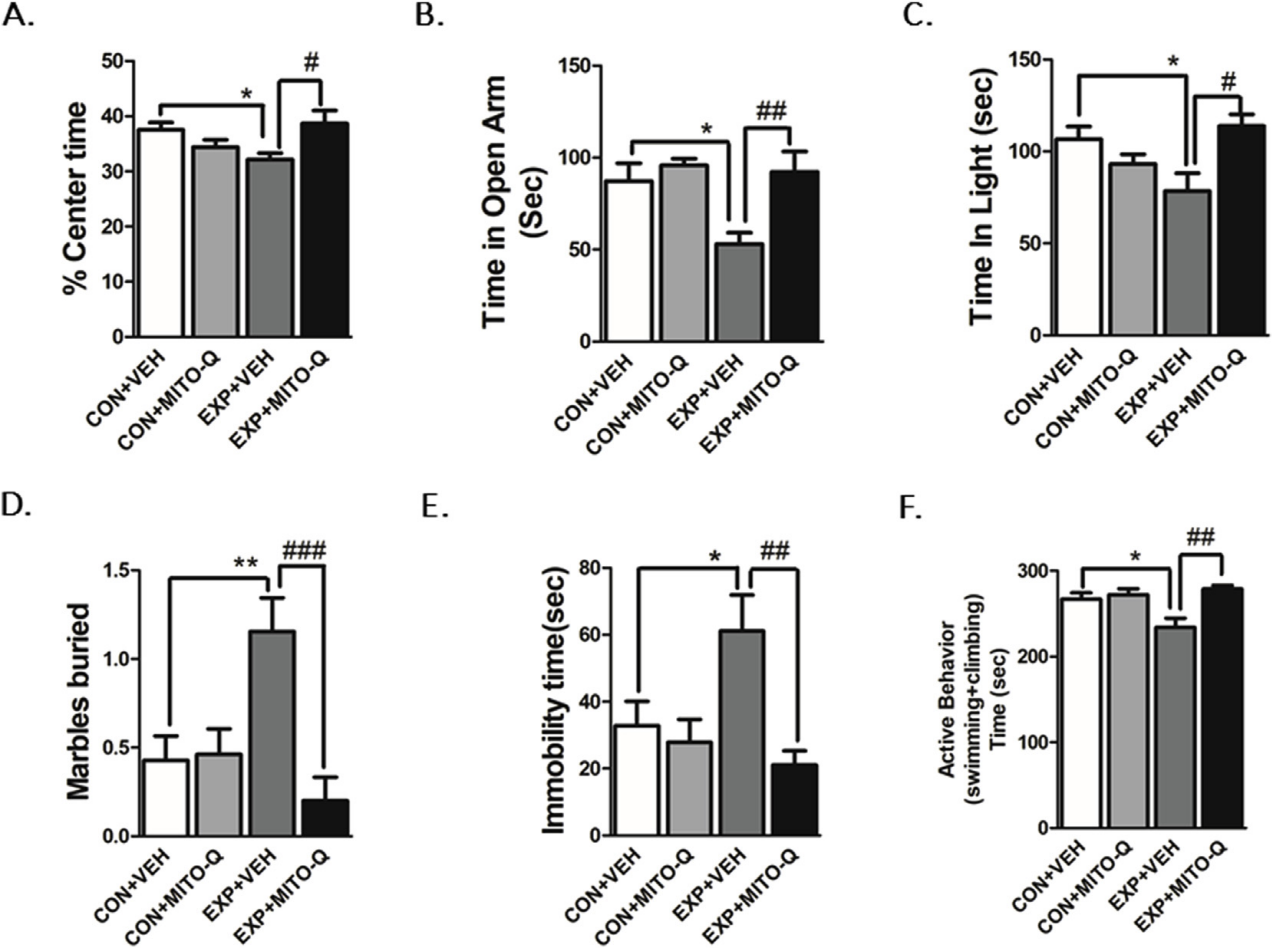
**Examination of anxiety-like behavior** in the (A) open field test (OFT), (B) Elevated Plus Maze Test, (C) Light-Dark Test and (D) Marble Burying Test in rats exposed to normal air/simulated vehicle exhaust with/without Mito-Q pre-treatment. **Examination of depression-like behavior** in the Force Swim Test by measuring (E) Immobility time and (F) Active behavior time in rats exposed to normal air/simulated vehicle exhaust with/without Mito-Q pre-treatment. (*) p < 0.05, (**) p < 0.01 significantly different from CON + VEH; (#) p < 0.05, (##) p < 0.01, (###) p < 0.001 significantly different from EXP + VEH; Values are mean ± SEM, n = 13 rats/group.

OFT indicated that EXP + VEH rats spent significantly less time in the center of the arena and more time in the periphery as compared to CON + VEH rats indicating an increased-anxiety-like behavior following SVEE (F_3,41_ = 3.881, P = 0.0157, Fig. 2A). No significant difference was observed in other OFT parameters such as total distance travelled and ambulatory activity, suggesting no alteration in locomotor activity of rats following SVEE. A similar observation was made in EPM test where EXP + VEH rats spent less time in the open arms (F_3,38_ = 6.821, P = 0.0009, Fig. 2B); and LD test where EXP + VEH rats spent less time in the lit compartment (F_3,36_ = 3.743, P = 0.0194, Fig. 2C). There was no significant difference observed in number of crossings in both EPM and LD test. In the MB test, EXP + VEH rats buried significantly more number of marbles (F_3,46_ = 6.845, P = 0.0007, Fig. 2D). All these measures are suggestive of SVEE-induced increased anxiety-like behavior. EXP + MITO-Q rats did not show these alterations indicating that Mito-Q treated rats were protected against SVEE-induced increase in anxiety-like behavior.

#### 4.2.2 Depression-like behavior

FST indicated that EXP + VEH rats spent significantly more time immobile as compared to CON + VEH rats indicating increased depression-like behavior following SVEE (F_3,45_ = 4.815, P = 0.0054, Fig. 2E). However, this behavior was not observed in EXP + MITO-Q rats, suggesting protective effect of Mito-Q in preventing SVEE-induced depression-like behavior. Similarly, EXP + VEH rats were observed to be engaged for a longer duration in active behavior comprising of swimming and climbing as compared to CON + VEH rats; and EXP + MITO-Q rats were protected from this behavior (F_3,44_ = 6.218, P = 0.0013, Fig. 2F).

### 4.3 Biochemical analysis

#### 4.3.1 Oxidative stress parameters

##### 4.3.1.1 8-Isoprostane levels

**Fig. 3:**
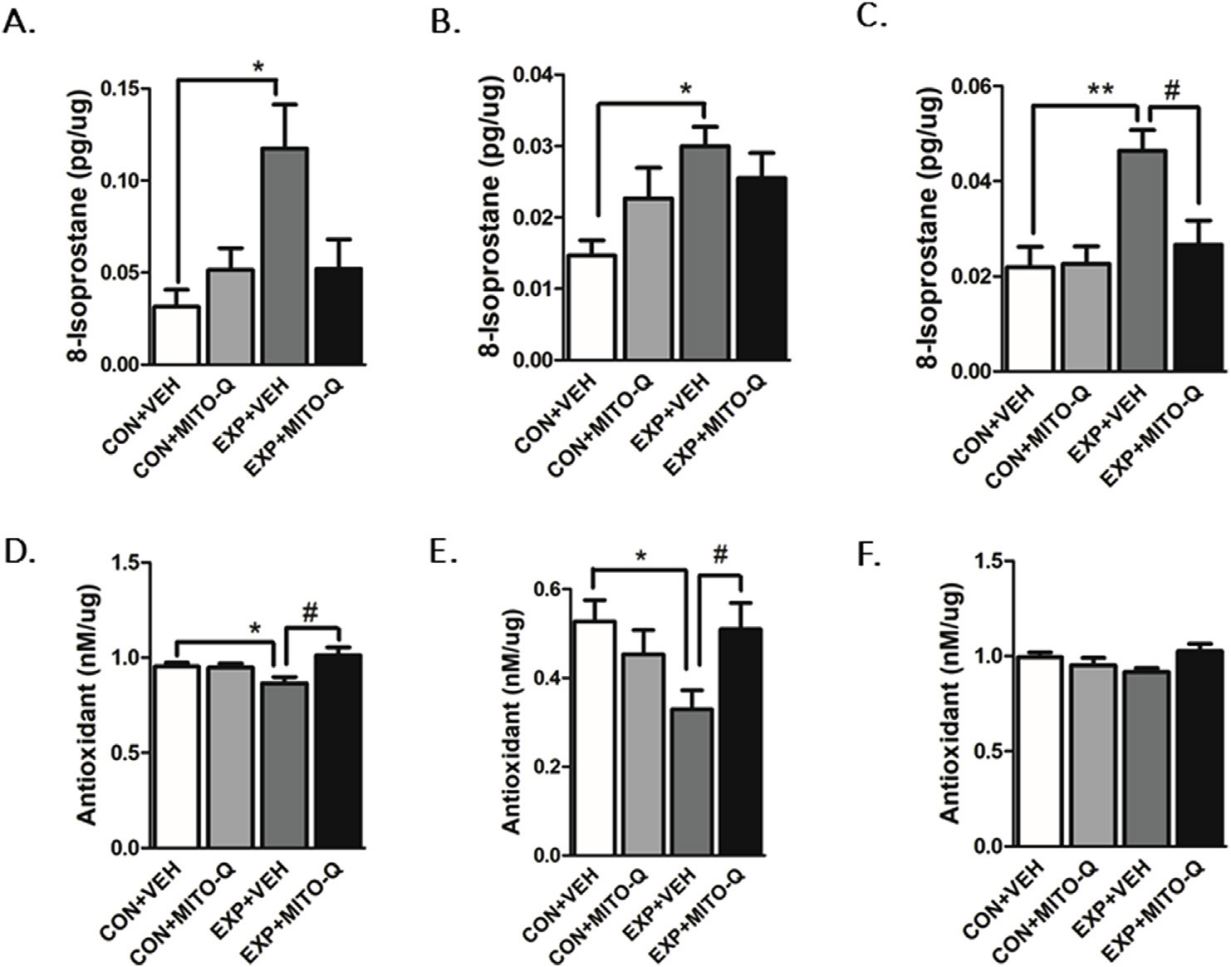
**Examination of oxidative stress levels.** Measurement of 8-isoprostane levels in (A) PFC, (B) Hippocampus (C) Amygdala; Measurement of Total Antioxidant Capacity in (D) PFC, (E) Hippocampus (F) Amygdala in rats exposed to normal air/simulated vehicle exhaust with/without Mito-Q pre-treatment. (*) p < 0.05, (**) p < 0.01 significantly different from CON + VEH; (#) p < 0.05, significantly different from EXP + VEH; Values are mean ± SEM, n = 6–8 rats/group.

SVEE led to significant increase in 8-isoprostane levels (Fig. 3A–C) in PFC (F_3,14_ = 4.257, P = 0.0247), hippocampus (F_3,11_ = 3.654, P = 0.0477) and amygdala (F_3,18_ = 6.777, P = 0.0030) of EXP + VEH rats as compared to CON + VEH rats. However, 8-isoprostane levels were unaltered in amygdala region of EXP + MITO-Q rats suggesting that Mito-Q treatment prevented SVEE-induced increases in oxidative stress in selected areas of the brain.

##### 4.3.1.2 Total antioxidant capacity (TAC)

A decrease in the TAC of antioxidants in PFC (F3,15 = 3.936, P = 0.0296) and hippocampus (F3,29 = 3.062, P = 0.0437) of EXP + VEH rats as compared to CON + VEH rats was observed, indicating a decrease in TAC following SVEE. Antioxidant capacity was unaltered in EXP + MITO-Q rats suggesting protective effect of Mito-Q in preventing SVEE-induced decrease in TAC. This alteration in TAC was not observed in amygdala (Fig. 3D–FFig. 3).

##### 4.3.1.3 Protein expression of Mn SOD

**Fig. 4:**
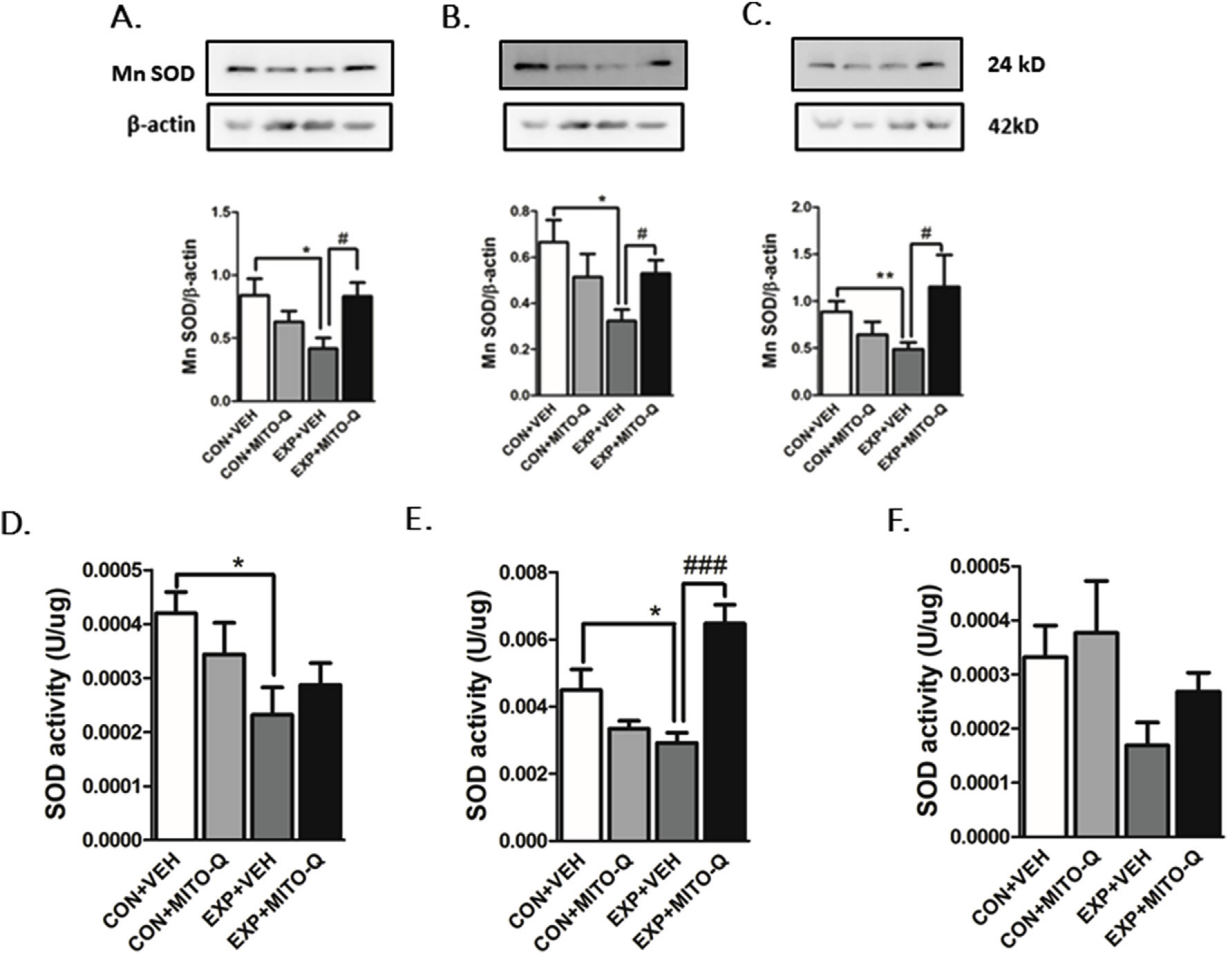
**Measurement of Mn SOD protein expression** in (A) PFC, (B) Hippocampus (C) Amygdala; and **Measurement of SOD activity** in (D) PFC, (E) Hippocampus (F) Amygdala in rats exposed to normal air/simulated vehicle exhaust with/without Mito-Q pre-treatment. Figures indicate cropped images of gels. (*) p < 0.05, (**) p < 0.01 significantly different from CON + VEH; (#) p < 0.05, (###) p < 0.001 significantly different from EXP + VEH; Values are mean ± SEM, n = 8–9 rats/group.

Protein expression of Mn SOD (Fig. 4A–C) was significantly lowered in PFC (F_3,12_ = 4.121, P = 0.0318), hippocampus (F_3,17_ = 3.210, P = 0.0494) and amygdala (F_3,21_ = 3.118, P = 0.0479) of EXP + VEH rats as compared to CON + VEH rats. However, Mn SOD expression did not change in EXP + MITO-Q rats, suggesting protective effect of Mito-Q in preventing SVEE-induced decrease in expression of Mn SOD.

##### 4.3.1.4 SOD activity

SVEE led to decrease in SOD activity in PFC (F_3,27_ = 3.012, P = 0.0474) and hippocampus (F_3,13_ = 12.53, P = 0.0004) of EXP + VEH rats as compared to other groups, however significant changes were not observed in the amygdala. SOD activity was unaltered in hippocampus of EXP + MITO-Q rats indicating that Mito-Q prevented decrease in SOD activity specifically in hippocampus (Fig. 4D–F).

#### 4.3.2 Mitochondrial parameters

##### 4.3.2.1 Mitochondrial oxygen consumption

**Fig. 5:**
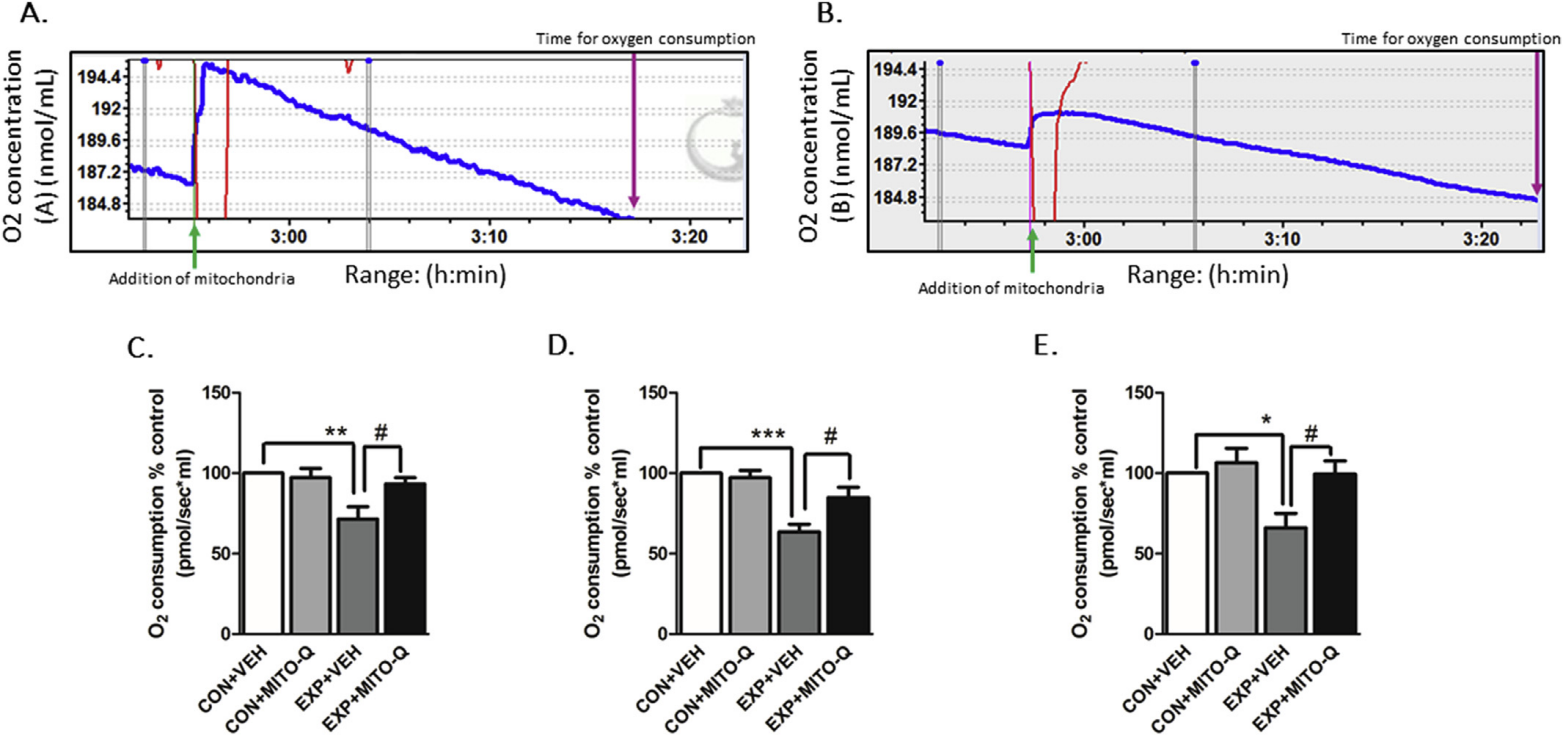
**Measurement of mitochondrial oxygen consumption.** Representative oxygraphs showing respiratory activity in (A) healthy and (B) damaged mitochondria. The rate of oxygen consumption is shown in pmol/sec*mL and represented as % control in mitochondria isolated from (C) PFC, (D) Hippocampus (E) Amygdala of rats exposed to normal air/simulated vehicle exhaust with/without Mito-Q pre-treatment. (*) p < 0.05, (**) p < 0.01, (***) p < 0.001 significantly different from CON + VEH; (#) p < 0.05 significantly different from EXP + VEH; Values are mean ± SEM, n = 6 rats/group.

Fig. 5A and B indicate oxygraphs depicting difference in oxygen consumption between healthy and damaged mitochondria. High resolution respirometry on mitochondria isolates of the brain regions indicated that SVEE led to decrease in oxygen consumption by the mitochondria of PFC (F_3,17_ = 7.577, P = 0.0020), hippocampus (F_3,17_ = 14.14, P < 0.0001) and amygdala (F_3,17_ = 6.028, P = 0.0055) of EXP + VEH rats as compared to other groups (Fig. 5C–E). Oxygen consumption remained unaltered in EXP + MITO-Q rats suggesting that rats that received Mito-Q were protected against SVEE-induced ETC disruption and subsequent decrease in mitochondrial oxygen consumption.

##### 4.3.2.2 ATP synthesis

**Fig. 6:**
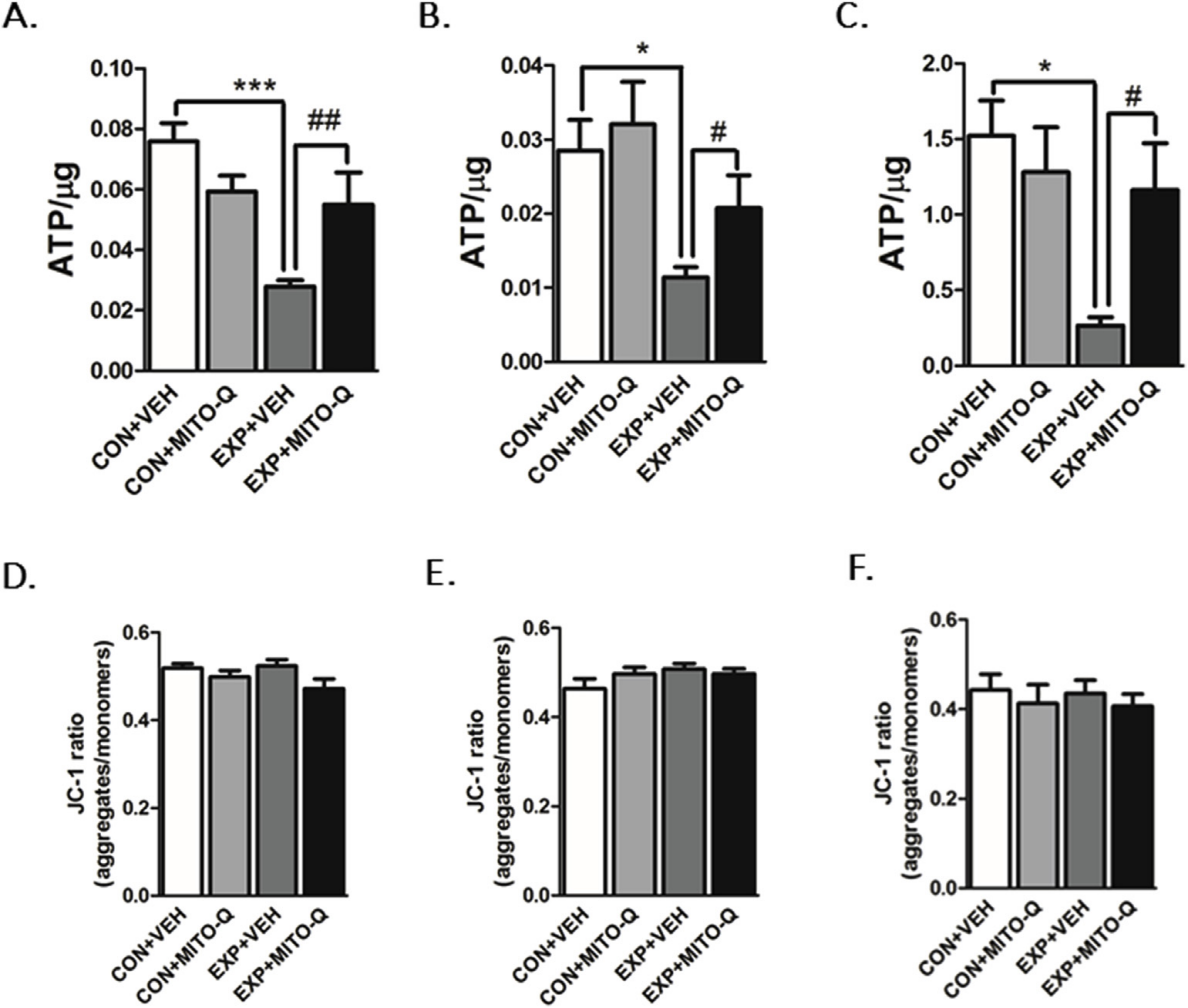
**Measurement of ATP synthesis** in (A) PFC, (B) Hippocampus (C) Amygdala; **Measurement of mitochondrial membrane potential** in (D) PFC, (E) Hippocampus (F) Amygdala in rats exposed to normal air/simulated vehicle exhaust with/without Mito-Q pre-treatment. (*) p < 0.05, (***) p < 0.001 significantly different from CON + VEH; (#) p < 0.05, (##) p < 0.01 significantly different from EXP + VEH; Values are mean ± SEM, n = 6–8 rats/group.

Luminometric ATP assay indicated that ATP levels were significantly reduced in PFC (F_3,31_ = 10.35, P < 0.0001), hippocampus (F_3,23_ = 4.639, P = 0.0112) and amygdala (F_3,15_ = 4.786, P = 0.0156) of EXP + VEH rats as compared to other groups, suggesting SVEE-induced decrease in ATP synthesis. This was contrary to EXP + MITO-Q rats, where ATP levels were comparable to control groups (Fig. 6A–C). This suggests that SVEE-induced lowering of ATP synthesis was prevented by Mito-Q pre-treatment.

##### 4.3.2.3 Mitochondrial membrane potential

JC-1 assay for measurement of membrane potential indicated that SVEE did not alter mitochondrial membrane potential in the brain regions, suggesting that SVEE-induced mitochondrial impairment does not affect membrane potential (Fig. 6D–F).

##### 4.3.2.4 Mitochondrial fission and fusion

**Fig. 7:**
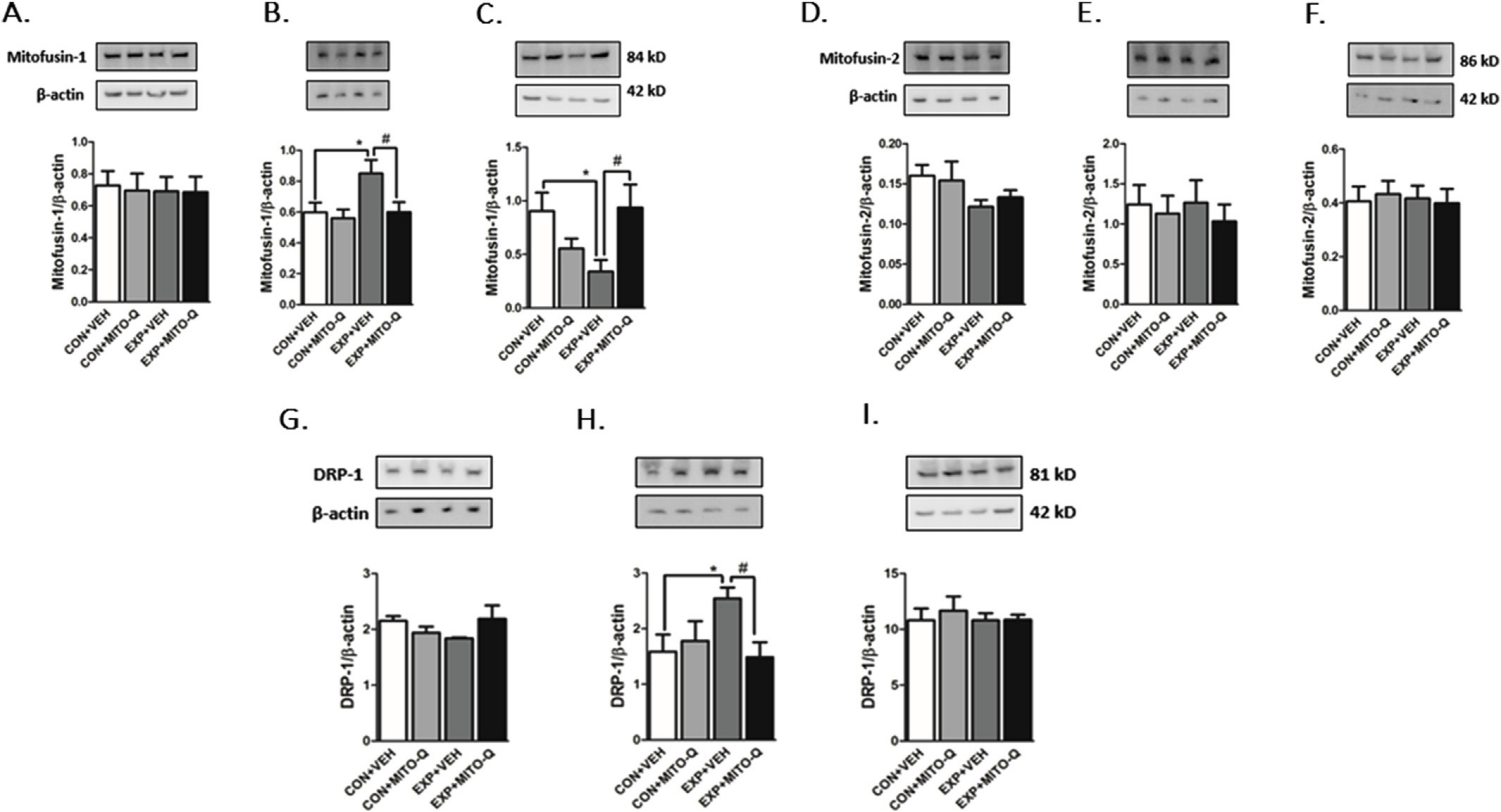
**Examination of mitochondrial fission and fusion.** Measurement of Mitofuin-1 protein expression in (A) PFC, (B) Hippocampus (C) Amygdala; Mitofusin-2 protein expression in (D) PFC, (E) Hippocampus (F) Amygdala; DRP-1 protein expression in (G) PFC, (H) Hippocampus (I) Amygdala, in rats exposed to normal air/simulated vehicle exhaust with/without Mito-Q pre-treatment. Figures indicate cropped images of gels. (*) p < 0.05 significantly different from CON + VEH; (#) p < 0.05 significantly different from EXP + VEH; Values are mean ± SEM, n = 9 rats/group.

Western blotting results indicated that SVEE led to increase in protein expression of fusion marker, mitofusin-1 in the hippocampus of EXP + VEH rats as compared to CON + VEH group (F_3,17_ = 3.424, P = 0.0410); whereas mitofusin-1 expression was decreased in the amygdala (F_3,14_ = 3.382, P = 0.0485) and remained unaltered in the PFC of EXP + VEH rats (Fig. 7A–C). On the other hand, mitofusin-2 expression did not alter significantly in either of the three brain regions. Mito-Q pre-treated rats were protected from alterations in Mfn-1 in hippocampus and amygdala (Fig. 7D–F).

SVEE led to increase in protein expression of fission marker, DRP-1 in the hippocampus (F_3,17_ = 3.286, P = 0.0462) and remained unaltered in PFC and amygdala of EXP + VEH rats (Fig. 7G–I). Mito-Q pre-treated rats were protected from these alterations in the hippocampus. This suggests that SVEE-induced mitochondrial impairment altered mitochondrial fission and fusion in a region dependent manner.

## 5 Discussion

Vehicle exhaust emissions are toxic constituents that act as both physical and emotional stressors (Thomson, 2019). Prolonged exposure to pro-oxidant constituents of vehicle exhaust such as CO_2_, CO and NO_2_ creates a toxic environment in the body that lead to buildup of ROS and RNS leading to oxidative stress (Westerholm and Egeback, 1994). (Betteridge, 2000). Vehicle exhaust-induced stress response via activation of HPA axis is another mechanism that is reported to result in oxidative stress and inflammation (Thomson, 2019). Similar to these reports, our preliminary work indicated that rats exposed to SVEE elicit elevated levels of corticosterone, a critical biomarker of systemic stress and HPA axis activation in the body (Supplementary fig. 3). The brain is considered highly vulnerable to oxidative stress, primarily due to its high oxygen consumption and lipid content, which causes excessive production of ROS (Salim, 2017). Psychological distress due to continuous exposure to exhaust constituents further aggravates this stressful environment (Sass et al., 2017).

Table 1 summarizes observed SVEE-induced behavioral and biochemical impairments and the protective role of Mito-Q against these deleterious effects. We observed that SVEE resulted in increased levels of oxidative stress in the PFC, hippocampus and amygdala regions of the brain, reflected from elevation in the levels of 8-isoprostane, a by-product of lipid peroxidation and a biomarker of oxidative stress (Birben et al., 2012). This observation was also supported by decrease in total antioxidant capacity and declining superoxide dismutase activity. The intensity of alterations in oxidative stress levels varied amongst different brain regions and could be attributed to differences in oxidative damage and inherent expressions of oxidative stress markers in different brain regions (Wang and Michaelis, 2010). Elevation of ROS and consequent neurotoxicity are linked to several neuropathologies and psychological disorders such as anxiety, depression and memory impairments (Patki et al., 2015; Allam et al., 2013; Salim et al., 2011; Solanki et al., 2015; Vollert et al., 2011; Rammal et al., 2008; Reckziegel et al., 2011). And, disruption in the HIP-AMY-PFC network is associated with behavioral deficits (Allam et al., 2013; Preston and Eichenbaum, 2013; Phelps and LeDoux, 2005; Courtney et al., 1998; Shin and Liberzon, 2010). Therefore, SVEE-induced rise in oxidative stress in the brain could be a causal force behind SVEE-associated behavioral impairments. Surprisingly, contrary to the observations in the brain, systemic levels of oxidative stress remained unaltered. One possible explanation for this observation could be the compensatory mechanisms operating at systemic levels that could have attenuated SVEE-associated increase in oxidative stress. Since the brain is highly susceptible to oxidative stress, the extensive damage inflicted from the rise in oxidative stress perhaps exceeds the repair and rescue effects of the antioxidant defense system, resulting in an unabated rise in oxidative stress and build-up of toxic environment in the brain.

**Table 1:**
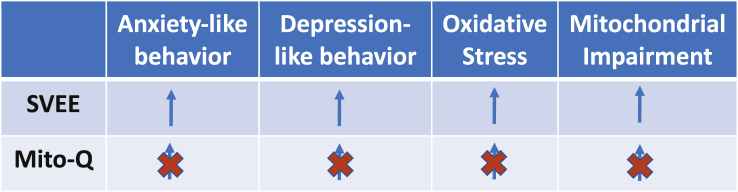
Summary of SVEE-induced behavioral and biochemical effects and protective effect of Mito-Q.

Build-up of ROS often leads to mitochondrial impairment, and inflammation thereby resulting in cellular dysfunction (Reuter et al., 2010; Newsholme et al., 2016). Relevant to this, we observed elevated levels of mitochondrial oxidative stress followed by impaired energy metabolism in isolated mitochondria from the PFC, hippocampus and the amygdala regions of SVEE rats. Mitochondria are highly vulnerable to oxidative stress owing to continuous ATP generation that involves transfer of electrons across the ETC followed by redox reactions (Huttemann et al., 2007). Leakage of electrons across these respiratory units leads to oxidation of free oxygen molecules thereby generating ROS and free radicals in the mitochondria (Santidrian et al., 2013). Mitochondrial antioxidant defense systems that primarily comprises of manganese superoxide dismutases (Mn SODs) are compromised in response to increase in oxidative stress levels, contributing to excessive buildup of ROS (Sarsour et al., 2010). SVEE rats exhibited reduced Mn SOD expression, thus leading towards a state of high mitochondrial oxidative stress. Mitochondrial ROS disrupt the ETC, lower oxygen consumption and hamper ATP synthesis resulting in mitochondrial impairment and cellular dysfunction. In extreme situations, mitochondrial impairment lowers mitochondrial membrane potential increasing mitochondrial membrane permeability and activation of apoptotic markers such as caspases eventually leading to cell death (Galluzzi et al., 2009; Druskovic et al., 2006). We observed a decrease in oxygen consumption accompanied with reduced ATP synthesis following SVEE, indicating ETC disruption and mitochondrial impairment. Surprisingly, mitochondrial membrane potential remained unaltered, suggesting that mitochondrial membrane integrity was not compromised. On the contrary, SVEE led to alteration in protein expression of fusion proteins- Mfn-1 and Mfn-2 as well as fission protein- DRP-1.

Mitochondrial dysfunction often triggers fission and fusion pathways in the mitochondria (Youle and van der Bliek, 2012; van der Bliek et al., 2013). Mitochondrial fusion is a compensatory mechanism that gets activated in response to increase in oxidative stress and results in fusion of two or more mitochondria prolonging mitochondrial sustenance (Chan, 2006). Therefore, alteration in expression of the dynamin proteins-Mfn-1/2 that mediates fusion in the mitochondria, is an indication of mitochondrial fusion. On the contrary, mitochondrial fission as a result of heightened oxidative stress split the mitochondria facilitating degradation (mitophagy) and cell death (Wu et al., 2011). Interestingly, an increase in expression of both fusion protein- Mfn-1 and fission marker- DRP-1 was observed in the hippocampus following SVEE, suggesting mitochondrial dysfunction. PFC and amygdala exhibited either decrease or unaltered expression of fusion and fission markers, suggesting a relatively lower mitochondrial dysfunction in this region. The reasons behind differences in the extent of mitochondrial damage in different brain regions is unclear. Our postulation is that variation in mitochondrial damage in different brain areas may be linked to differential vulnerability of different brain regions to oxidative stress. Relevant to this, it has been reported that brain regions differ in their susceptibility to oxidative stress, owing to differences in intrinsic oxidative stress levels, demand for ROS/RNS-based signaling and onset of inflammatory responses (Wang and Michaelis, 2010). Therefore, although we observed SVEE-induced oxidative damage in all brain regions, the level of oxidative stress in a particular brain area, determined the extent of mitochondrial damage and consequent activation of trigger fission-fusion pathways. Thus, hippocampus with greatest level of oxidative stress also exhibited greatest mitochondrial damage among the three brain areas. Moreover, expression of mitophagy marker, parkin remained unaltered in all the three brain regions in response to SVEE, suggesting that SVEE-induced mitochondrial impairment did not result in its degradation (data not shown). It is reasonable to suggest that SVEE-induced oxidative stress led to mitochondrial and cellular dysfunction. Mitochondria play a critical role in neuroplasticity and calcium homeostasis that controls energy metabolism in the brain (Kato, 2006; Salminen et al., 2015). And, mitochondrial impairment is believed to alter neuroplasticity and impair transmission of the neural networks; and has been linked to anxiety and depressive disorders (Bansal and Kuhad, 2016; Modica-Napolitano and Renshaw, 2004; Lin and Beal, 2006). Therefore, it is plausible that elevated levels of oxidative stress and mitochondrial impairment are the causal link between SVEE and behavioral deficits.

To confirm this causal link between SVEE, oxidative stress and behavioral deficits, we treated rats with a mitochondria-specific antioxidant Mito-Q prior to SVEE in order to determine its preventive effect. Mito-Q, is a semi-synthetic lipophilic derivative of ubiquinol, one of the endogenous antioxidants that play a critical role in the body (Murphy and Smith, 2007; Mao et al., 2013). It is lipophilic nature and a positively charged group attached to its parent structure increases its accumulation to higher concentrations in the mitochondria (Murphy and Smith, 2007). To confirm the accumulation of Mito-Q in the brain, we performed LC-MS quantification, which suggested that although Mito-Q was administered via oral route, it crossed the blood brain barrier and accumulated in the brain regions of interest. We observed that rats receiving Mito-Q pre-treatment were protected from SVEE-induced behavioral, oxidative stress and mitochondrial impairment. However, the degree of protective action of Mito-Q against oxidative stress and mitochondrial fission-fusion varied between different brain regions. For instance, Mito-Q did not completely prevent rise in 8-isoprostane levels in the PFC and hippocampus and was not entirely effective in preventing decrease in SOD activity or alteration in DRP-1 expression in PFC and amygdala. One probable explanation for this could be that the antioxidant action of Mito-Q is not equally effective in all brain regions and not adequate for mitigating oxidative stress. Also, since Mito-Q primarily acts by scavenging peroxyl radicals (McManus et al., 2011), it is possible that it reduces overall oxidative stress, but is not effective enough to protect expression and activity of specific antioxidants.

In conclusion, our study demonstrates that SVEE is a significant physical and psychological stressor that leads to behavioral deficits in rats. We further established that SVEE-induced oxidative stress results in mitochondrial impairment, which most likely is the mechanism responsible for alteration of neural circuitry, which regulates cognitive and behavioral functions. The intensity and extent of these impairments, and the level of damage in the circuitry remains to be investigated.

## Declaration of competing interest

The author(s) declare no competing interests.
