# Supplementary material for: Involvement of oxidative stress and mitochondrial mechanisms in air pollution-related neurobiological impairments
Source: Neurobiol Stress. 2019 Dec 19;12:100205. doi: 10.1016/j.ynstr.2019.100205 (PMC7109516; doi:10.1016/j.ynstr.2019.100205)
Supplement: Multimedia component 1 [file mmc1.pdf]

Supplementary Figures

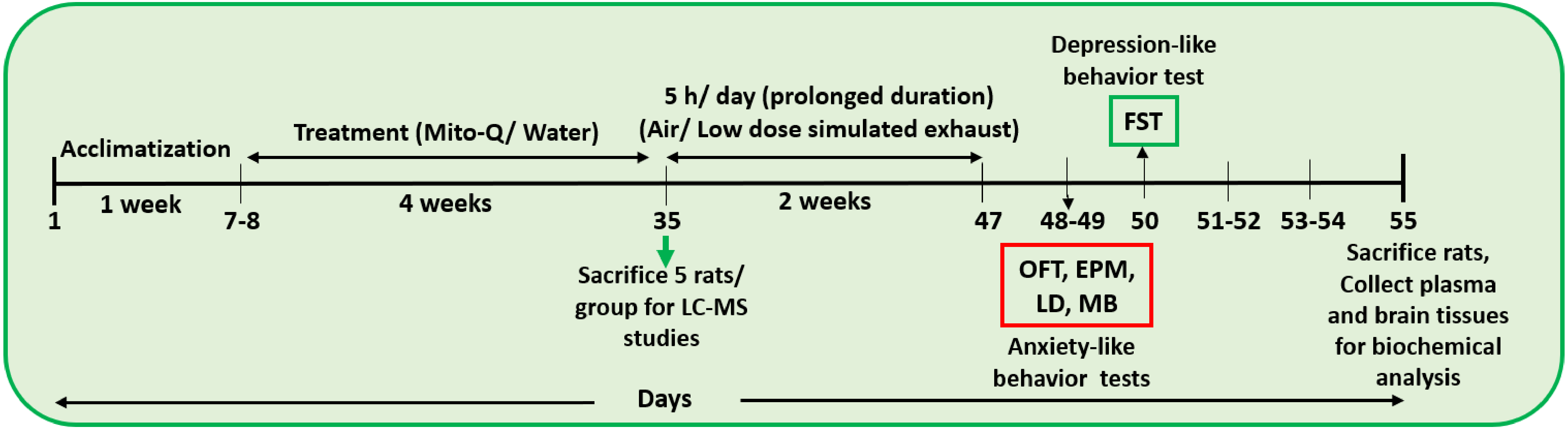

Supplementary Figure 1. Experimental Design: experimental scheme followed to conduct simulated vehicle exhaust exposures in Sprague Dawley rats.

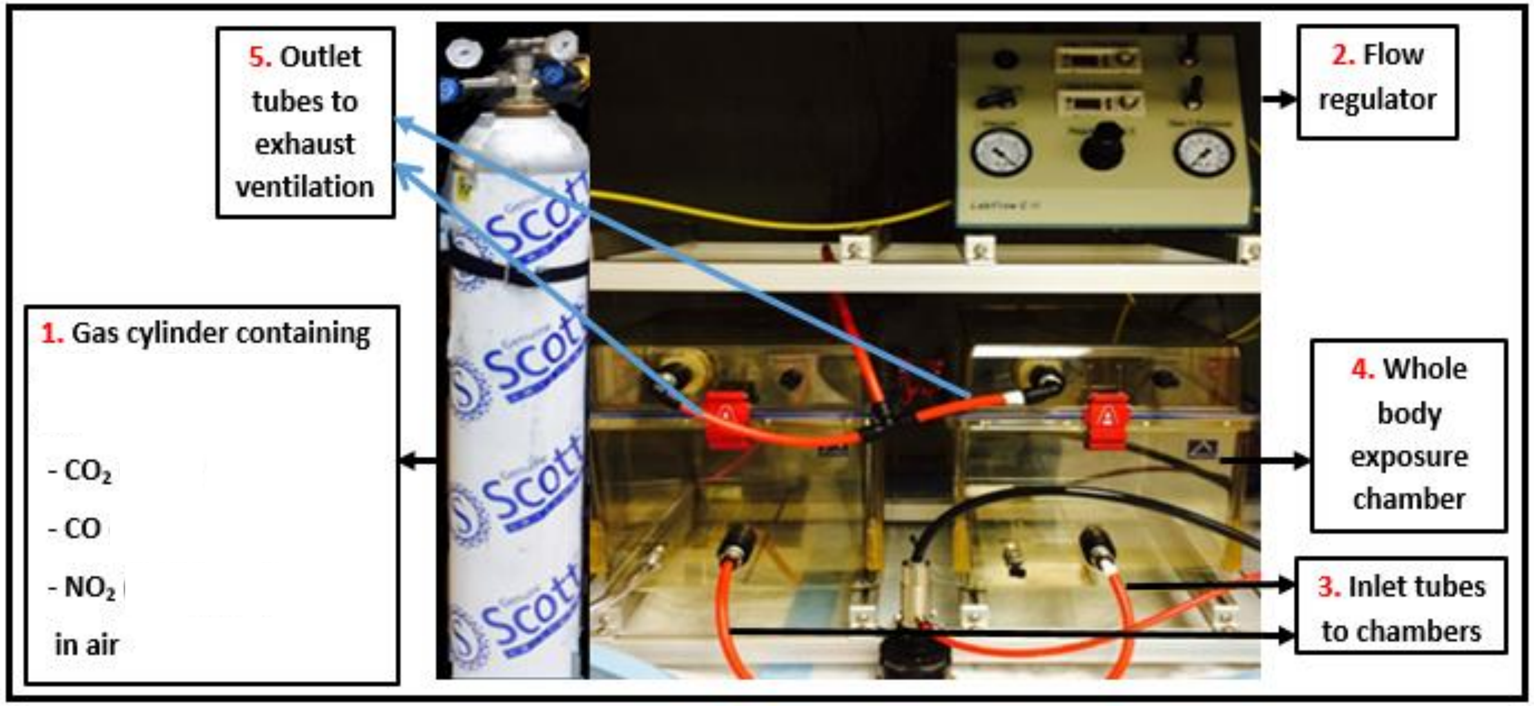

Supplementary Figure 2. Simulated Vehicle Exhaust Exposure (SVEE) Apparatus: air-tight apparatus used to conduct whole body exhaust exposures in Sprague Dawley rats. Simulated vehicle exhaust from the gas cylinder was modulated using the flow regulator into inlet tubes that entered exhaust chambers. Inlet and outlet tubes maintained a continuous circulation into the chambers.

3A. 3B. 3C.

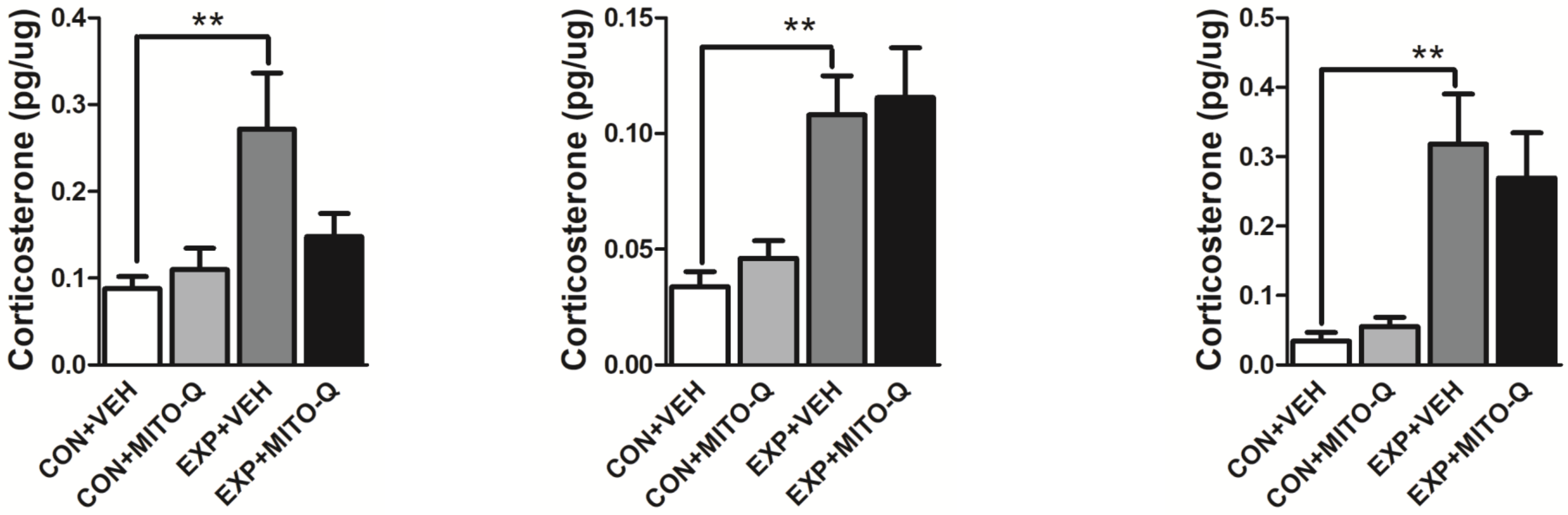

Supplementary Figure 3. Measurement of corticosterone levels in (A) PFC ($F_{3,17}$=5.334, P=0.0090), (B) Hippocampus ($F_{3,16}$=8.560, P=0.0013) (c) Amygdala ($F_{3,17}$=9.021, P=0.0008) in rats exposed to normal air/ simulated vehicle exhaust with/without Mito-Q pre-treatment. (**) p< 0.01, significantly different from CON+VEH; Values are mean ± SEM, n=6 rats/group
